# Posterior reversible encephalopathy syndrome case report in an untreated, normotensive, ovarian cancer patient in the presence of paraneoplastic antibodies

**DOI:** 10.1186/s12883-020-01913-y

**Published:** 2020-09-02

**Authors:** Elad Barber, Rijini Nugzar, Vitaly Finkelshtein, Alexander Puzhevsky, Tally Levy

**Affiliations:** 1 Division of Gynecologic Oncology, Wolfson Medical Center, Holon, Israel; 2 Department of Obstetrics and Gynecology, The Edith Wolfson Medical Center, P.O. Box 5, 58100 Holon, Israel; 3 Sackler School of Medicine, Tel Aviv University, Tel Aviv, Israel; 4 Department of Anesthesia, Wolfson Medical Center, Holon, Israel; 5 Department of Neurology, Wolfson Medical Center, Holon, Israel; 6 Department of Diagnostic Radiology, Wolfson Medical Center, Holon, Israel

**Keywords:** Posterior reversible encephalopathy syndrome, PRES, Paraneoplastic antibodies, CRMP5, Ovarian cancer

## Abstract

**Background:**

Posterior reversible encephalopathy syndrome (PRES) is a rare neurological condition with many associated risk factors. The presentation varies and consists of seizures, impaired visual acuity or visual field deficits, disorders of consciousness, headaches, confusion and focal neurological deficits. The diagnosis relies on clinical presentation and MRI findings. Treatment and prognosis are related to the underlying etiology.

**Case presentation:**

We present a 58-year-old woman with ovarian cancer who developed symptoms and radiologic signs of PRES with no apparent trigger other than a sudden increase in blood pressure for the first time in her life and before any treatment has begun. Antibodies to collapsin response-mediator protein-5 (CRMP-5), a malignancy related paraneoplastic protein, were identified in her CSF.

**Conclusions:**

We present a novel and intriguing association between PRES and antibodies against CRMP-5 which may highlight a new etiology for this condition.

## Background

Posterior reversible encephalopathy syndrome (PRES) is an acute, rare, reversible, neurological condition and is characterized by a variety of symptoms including seizures, impaired visual acuity or visual field deficits, disorders of consciousness, headaches, confusion and focal neurological deficits [1].

The main speculated etiology is hypertension causing failed autoregulation and hyper-perfusion consequentially leading to vascular cerebral dysregulation [2, 3]. Other PERS associated risk factors have also been shown to affect the capillary system. Immunosuppression and chemotherapy have been shown to alter capillary morphology [4, 5]. Malignant tumors can cause activation of endothelial cells, proliferation and neovascularization mainly through the vascular endothelial growth factor (VEGF) family influencing endothelial motility and leading to neovascularization [4].

The prompt diagnosis of atypical presentations of PRES is important to avoid delays in diagnosis and treatment, as is identification of complicating factors which may adversely affect patient prognosis. Moreover, treatment of PRES depends on the underlying etiology along with antihypertensive and antiepileptic therapy, when needed [6]. Consequently, it is important to study and better characterize the predisposing factors that can bring about the appearance of this condition.

The diagnosis of PRES consists of an appropriate clinical presentation of neurological symptoms mostly of headache, visual disturbances confusion and seizures. Suitable brain MRI features consist of posterior subcortical vasogenic edema, hyperintense signals on T2-weighted images and FLAIR [7]. EEG findings are not specific for the condition and cannot assist to a good extent in affirming the diagnosis [8].

Two case reports have described PRES in ovarian cancer patients [9, 10]. Both were associated with prior treatment with chemotherapy. In one case, the patient was treated with neoadjuvant Carboplatin and Paclitaxel chemotherapy prior to onset [10]. The second patient was treated with Bevacizumab (Avastin) [9]. These chemotherapeutic agents have been known to be associated with PRES [2, 9, 10].

In the following report, we present a woman with ovarian cancer who developed symptoms and radiologic signs of PRES prior to any treatment. An association to paraneoplastic antibodies is suggested.

## Case presentation

A 58-year-old woman with ovarian cancer was electively admitted to our gynecologic oncology division for surgical debulking. CT showed a right ovarian tumor measuring 2.2X2.6 cm, ascites and an omental mass. The uterus and left ovary appeared normal. Her CA-125 level was 233 U/mL. CT-guided-biopsy from the omentum revealed high-grade-serous-carcinoma. Her past medical history included anxiety and fibromyalgia treated with serotonin reuptake inhibitors (SRI’s) and Tramadol. She had no other medical, family and psycho-social history including relevant genetic information.

Upon admission, she had no complaints with normal physical-examination and lab work. Upon entrance to the operating room (OR) and before any procedures were performed, she started to demonstrate convulsions in the face and rigidity accompanied by foam from the mouth and irregular eye movement. This episode lasted 10 min and was accompanied by increased blood pressure (maximal measurement of 190/100) and bradycardia. The blood pressure and bradycardia normalized within several minutes without any treatment.

As soon as the patient resumed consciousness, she complained of blindness, severe headache and appeared confused. A neurological examination performed in the OR demonstrated a positive bilateral Babinski sign as the only pathological finding.

Ophthalmologic and psychiatric evaluations were normal. Brain CT was normal with no signs of active bleeding or mass. During the hours that followed the acute onset, there was further deterioration in her confusion and blindness and the patient reported continuation of the severe headache which did not respond to analgesia. In addition, fever was measured (38.7 °C). The only abnormal laboratory finding was an elevated white blood cell (WBC) count of 12,200 10^3^/μL with 93% polymorphonuclear cells (PMNs(.

Due to the possible diagnosis of meningitis/encephalitis, an empiric Acyclovir treatment was begun and lumbar puncture (LP) was done. The LP fluid was clear, with an opening pressure of 201 mmH2O, increased WBC count (37 cells/mm^3^, mostly PMN’s) increased protein count (125 mg/dl) with normal glucose level (72 mg/dl) and a decreased chloride level (123 mmol/L). Culture from the CSF was negative. In addition, since there was uncertainty regarding the origin of seizures, Valproic acid was initiated. EEG showed a non-specific sign of left temporal irregularity. No epileptic activity was demonstrated.

**Fig 1:**
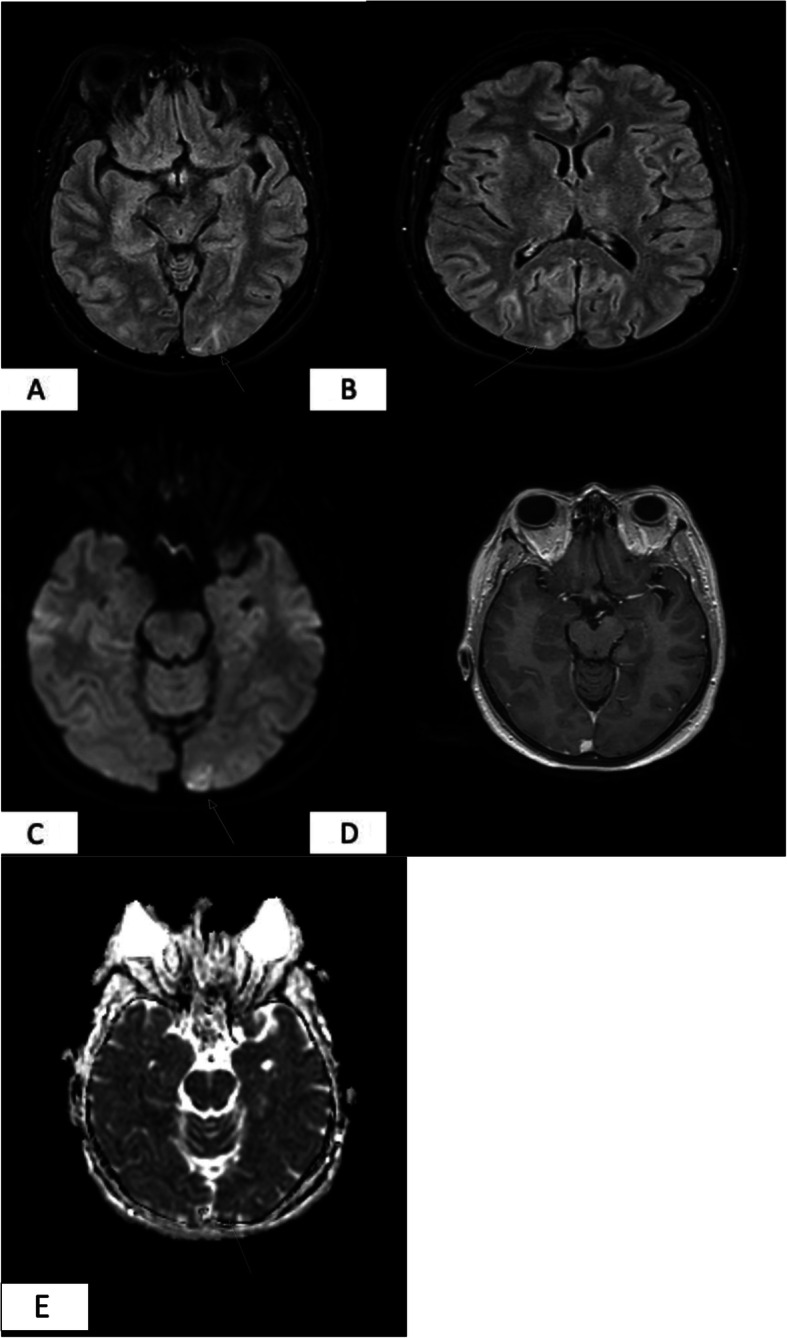
**a+b**: Axial FLAIR image showing cortical hyperintensity in both occipital lobes. **a**- Left; **b**- Right; **c**. DWI image showing restricted diffusion in left occipital cortex; **d** Axial T1 post Gadolinium image showing no pathological enhancement. **e**- ADC map showing mild diffusion restriction

Brain MRI including T1, T2, fluid-attenuated inversion recovery (FLAIR), diffusion weighted imaging (DWI), apparent diffusion coefficient (ADC) and susceptibility weighted imaging (SWI) sequences were performed before and after Gadolinium administration. Occipital cortical and subcortical T2 hyperintense signals were observed mainly on the left side (Fig.1 a) and, to a lesser extent, on the right side (Fig. 1b). There was moderate hyperintensity on DWI in the left occipital cortex (Fig. 1c). There was no abnormal enhancement after Gadolinium administration (Fig. 1d). Mild diffusion restriction was demonstrated on the ADC maps (Fig. 1e).

Polymerase chain reaction (PCR) for Herpes simplex virus, Varicella zoster virus and Enterovirus were negative. Urine and blood cultures were negative. Chemistry including creatinine kinase, liver function tests and electrolytes were normal. Urine toxicology yielded no abnormal findings.

During her hospitalization, the patient demonstrated a continuous improvement. Repeat neurological examination showed complete consciousness, intact visual fields and no neurological deficits. Virology results came back negative excluding viral encephalitis. No other treatments were given, including steroids.

As it is known that seizures can be a manifestation of paraneoplastic syndromes, specifically, paraneoplastic epilepsy, a panel of tests for the detection of paraneoplastic syndromes were obtained from the patient’s blood and CSF fluid according to latest recommendations [11]. A neuronal autoimmune antibody (Ab) screen was performed including Abs against GABAB, NMDR, CASPR2, AMPAR1, AMPAR2, LGl1, Amphiphysin, CRMP-5 (CV2), PNMA2, Ri, Yo, Hu, Recoverin, SOX1 and Titin (see abbreviations).

All tested antibodies in the CSF and serum came back negative except for CRMP-5 Abs which tested positive in the CSF but was negative in the serum.

CRMP5 protein, also known as Neuronal CV2, is thought to be involved in neural development and antibodies to CRMP5 were found in some patients with paraneoplastic syndromes presenting with neurologic symptoms [12]. As antibodies against CRMP5 are known to be associated with small cell lung carcinomas and thymomas [12], a chest CT was performed in our patient and came back normal. All other tested autoimmune antibodies were negative.

In view of all the clinical presentation, imaging and laboratory results, the woman was diagnosed with PRES.

The patient was discharged with complete resolution of her symptoms. Repeat MRI one month later showed complete disappearance of the previously described radiologic findings. She was started on Paclitaxel and Carboplatin chemotherapy. After 3 chemotherapy cycles she was doing well with no neurological complaints. Abdominal CT showed good response to the chemotherapy and the patient underwent interval debulking including bilateral salpingo-oophorectomy, hysterectomy, omentectomy with transverse colectomy for complete cytoreduction. No neurological symptoms were observed prior or after the operation.

## Discussion and conclusions

Our patient’s symptoms of seizure, confusion, headache and blindness and the MRI findings of occipital cortical and subcortical T2 hyperintense signal and mild diffusion restriction in the left occipital cortex were all compatible with the diagnosis of PRES [13, 14]. No other brain lesions were observed on brain CT and MRI excluding brain metastases as the cause of the clinical symptoms. To our knowledge, this is the first report of PRES in a patient with ovarian malignancy with no other known risk factors for PRES such as previous diagnosis of hypertension, epilepsy, chemotherapy, or bevacizumab treatment.

Hadad and Billingsley [10] described PRES in an ovarian cancer patient. A paraneoplastic, autoimmune etiology was provided due to the presence of voltage-gated potassium channel antibodies, known to induce neurological symptoms. However, their patient also suffered from hypertension and was treated with chemotherapy, specifically gemcitabine, both known to be precipitating factors for PRES.

Other known risk factors for PRES are renal failure, immunosuppressant drugs, chemotherapy, eclampsia and autoimmune disorder [15]. None of these were present in our patient and all her blood tests including creatinine, calcium and albumin levels were within normal limits.

The only significant related factors were ovarian malignancy and the interesting finding of Abs to CRMP-5 in the CSF raising paraneoplastic syndrome as a possible etiology for PRES in our patient. CRMP-5 Abs are present in different disorders of the central and peripheral nervous systems including seizures and confusion [12] and were discovered in patients with small cell lung cancers (SCLC) and thymomas [16]. To date, there have been no reports of an association between antibodies against CRMP5 and the diagnosis of PRES. Moreover, no reports have been made about the association of CRMP-5 and ovarian cancer.

No CRMP-5 Abs were found in the serum, However, it is known that 15% of patients with paraneoplastic antibodies present with positive CSF titers and negative antibody levels in the serum [16], as was the case in our patient. In addition, according to the recommended diagnostic criteria for paraneoplastic neurological syndromes [17] the diagnosis of a paraneoplastic neurological syndrome is possible in cases of partially characterized onconeural antibodies, given an underlying diagnosis of cancer.

EEG findings associated with paraneoplastic epilepsy are usually nonspecific and consist mainly of generalized slowing and/or focal slowing and extratemporal abnormalities. Due to the non-specific findings in our patient’s EEG and taking into account the fact that EEG results cannot be relied on with regards to the diagnosis of either PRES or paraneoplastic epilepsy, this test did not help with the diagnosis.

The main mechanism behind PRES is thought to be a dynamic vascular change in two possible fashions [13]. First, a hyper-perfusion state may bring about a blood-brain barrier (BBB) breakthrough and consequentially, extravasation of fluid, resulting in cortical or subcortical edema. Second, vasospasm may be the underlying mechanism, which may evidently cause cytotoxic edema [13].

Several autoimmune anti-glutamate receptor antibodies which are also known as paraneoplastic proteins, i.e. anti–NMDR, anti-AMPR, anti-mGluR1 and anti-mGlur5 were found to activate BBB endothelial cells and induce neurological changes [18]. Recently, paraneoplastic optic neuritis, vitritis, retinitis and optic disk edema were described in CRMP-5 Ab positive patients indicating its role in central neurological signs [17]. In view of the finding of PRES in our patient and presence of the paraneoplastic anti-CRMP-5 in her CSF, it is suggested that this protein might activate endothelial cells as other paraneoplastic Abs.

Furthermore, Ovarian cancer is characterized by intense neovascularization formed by proliferation, migration and formation of tube-like structures by endothelial cells mainly under the influence of VEGF [5]. VEGF is also known to enhances vascular permeability and increases BBB leakage [18]. All these processes influencing endothelial cells and vascular permeability can explain the occurrence of PRES in our patient.

To conclude, our patient demonstrates a unique presentation of PRES. Finding of antibodies to CRMP-5, a malignancy related paraneoplastic protein may highlight a new etiology for PRES. It is suggested that every case of PRES should be thoroughly evaluated for the presence of autoimmune and paraneoplastic antibodies for early diagnosis and treatment of the underlying cause leading to PRES. Better characterization and understanding of this condition, its risk factors and the influence of paraneoplastic Abs on BBB should be explored further.

## Data Availability

All the data during our patient’s hospitalization are computerized and freely accessible and available from the corresponding author on request by the editor.

## Abbreviations

CRMP5: Collapsin response mediator protein (also known as CV2)
Anti-Hu: Anti-ANNA1 associated encephalitis
ANNA-2, “Anti Ri”: Antineuronal nuclear autoantibody type 2
PCA-1 (“Anti Yo”): Purkinje cell cytoplasmic antibody type 1
Anti SOX-1: Anti SRY-related HMG-box
Anti NMDR: Anti N-methyl-D-aspartate receptor
Anti Caspr2: Anti–contactin-associated protein-like 2
anti GABA-B: Gamma-aminobutyric acid receptor
anti AMPAR: Anti α-amino-3-hydroxy-5-methyl-4-isoxazolepropionic acid receptor
LGI1: Leucine-rich glioma-inactivated 1
PNMA2: Paraneoplastic antigen Ma2
